# Evaluation of Relationship between Lichen Planus and HCV Antibody

**DOI:** 10.5681/joddd.2010.003

**Published:** 2010-03-14

**Authors:** Ali Taghavi Zenouz, Masoumeh Mehdipour, Narges Gholizadeh, Behrooz Naghili, Mohammad Jafari Heydarlou

**Affiliations:** ^1^ Assistant Professor, Department of Oral Medicine, Faculty of Dentistry, Tabriz University of Medical Sciences, Tabriz, Iran; ^2^ Professor, Infectious and Tropical Diseases Research Center, Tabriz University of Medical Sciences, Tabriz, Iran; ^3^ Assistant Professor, Department of Oral Medicine, Faculty of Dentistry, Uremia University of Medical Sciences, Uremia, Iran

**Keywords:** Antibody, HCV, lichen planus

## Abstract

**Background and aims:**

Lichen planus is a relatively common chronic mucocutaneaous disease with an unknown cause, and is considered a manifestation of cell-mediated immune response. Hepatitis C virus (HCV) and its subgroups have been associated with lichen planus in different geographic locations. The present study was undertaken to evaluate the prevalence of HCV antibody in patients with lichen planus in northwest Iran.

**Materials and methods:**

This descriptive analytical study included 30 patients with cutaneous lichen planus, 30 pa-tients with oral lichen planus, and 30 healthy individuals as controls. Anti-HCV test was run for all the subjects. Descriptive statistics as well as chi-square test, to compare means in the three study groups, were applied to the data using SPSS 14.0 computer software.

**Results:**

Age and sex differences between the groups were not significant. No statistically significant differences were ob-served in anti-HCV test results between the groups (P = 0.50).

**Conclusion:**

No statistically significant relationships were observed between lichen planus and HCV antibody in the studied samples.

## Introduction

Oral lichen planus is a chronic inflammatory and immunologic mucocutaneaous lesion, typically affecting adults in the fifth decade of life with a female to male ratio of 3:2.^1^ The disease is rarely seen in children.^2^ Prevalence of cutaneous and oral lichen planus have been reported to be 0.2–1% and 0.1–2.3% in western countries, respectively.^1 , 3^ The disease can afflict any surface of the body, but it is mostly seen on the ventral aspect of wrists, on lumber areas and around ankles. Wrists and ankles are common sites for hypertrophic lesions. When palms of the hands and soles of the feet are affected, the lesions are rough and indurated with a yellow discoloration. Itching is a constant manifestation of lichen planus, ranging from occasionally mild itching episodes to relatively severe and constant episodes, which may interfere with sleeping and may disrupt normal course of life, making it intolerable. In some cases no itching is present.

No etiologic factors have been found for lichen planus. It has been reported that foreign antigens such as infectious diseases, medications, and chemical agents induce the disease through immunologic changes in the cell-mediated immunity and influence on HLA.^4^ Recently, more attention has been focused on the relationship between lichen planus and infections. Hepatitis C virus (HCV) infection not only afflicts liver, but it also has extra-hepatic manifestations such as oral lichen planus (especially erosive type) and oral cancer.^5^ HCV is an important factor in chronic hepatitis, which results in debilitation in 20-30% of the infected individuals, severe hepatic involvement, and death in some cases.^6^

Since HCV and its subgroups have been shown to have a suspected role in lichen planus in different geographic locations, the present study was undertaken to evaluate the prevalence of HCV antibody in lichen planus patients in northwest Iran.

## Materials and methods

In this descriptive, analytical study, 30 patients with cutaneous lichen planus, 30 patients with oral lichen planus, and 30 healthy individuals were consecutively selected from those referring to the Department of Oral Medicine at Faculty of Dentistry, Tabriz University of Medical Sciences, or to the Dermatology Clinic of Sina Hospital, Tabriz, Iran.

Diagnosis of oral lichen planus in all patients was confirmed by histopathological examination. The method for diagnosis of skin lesions was clinical evaluation. Patients with concurrent oral and cutaneous lichen planus were excluded from the study. None of the subjects in the three groups had any congenital or acquired defects in their immune systems, including AIDS; in addition, none of the subjects had any history of drug abuse, hemodialysis, blood transfusions or any history of receiving blood products.

The studied variables included age, gender, type of lichen planus, the location of the lesion, and the presence or absence of pain, burning sensation and itching, which were recorded in questionnaires designed for the purpose of this study. Subjects in all groups underwent HCV antibody test using the ELISA method.

Descriptive statistics was used to report the results. Chi-square test was also employed to compare means in the three groups studied using SPSS 14.0 computer software. Statistical significance was set at P < 0.05.

## Results

Means age in patients with oral lichen planus, cutaneous lichen planus and the controls were 40.16 ± 11.5, 39.7 ± 8.9, and 36.6 ± 11.8 years, respectively. Age differences between the three groups were not statistically significant (P = 0.44, f _(2,87)_ = 0.81).

**Figure 1:**
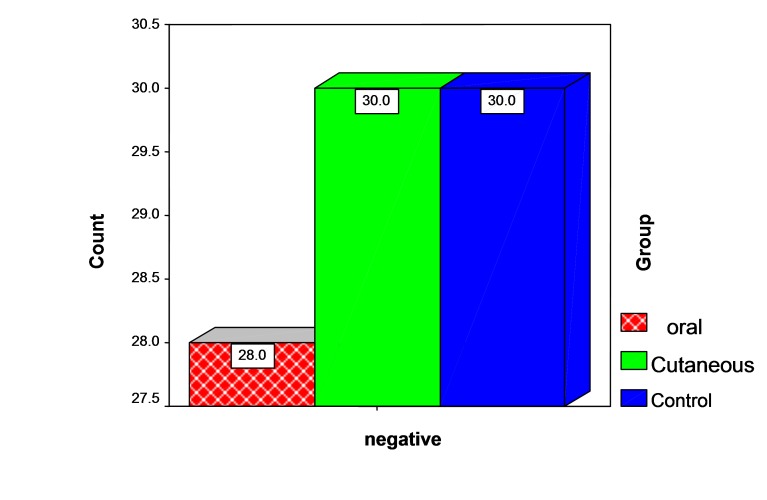
Frequency distribution of the results of HCV antibody tests in the three groups.

The oral lichen planus group consisted of 15 males (50%) and 15 females (50%); the cutaneous lichen plans group consisted of 17 males (56.7%) and 13 females (43.3%); and the control group consisted of 19 males (63.3%) and 11 females (36.7%). No statistically significant differences were observed between the groups regarding distribution of males and females in the groups (χ ^2^ = 1.08, df = 2, P = 0.58). All the subjects had negative results for HCV antibody. Figure 1 shows the results of HCV antibody tests.

## Discussion

Lichen planus is regarded as a premalignant lesion. Prevalence of squamous cell carcinoma (SCC) has been reported to be between 0.4% and 2% in various groups during a 5-year study period among lichen planus patients.^1^ A comprehensive study has reported the prevalence of SCC to be 1.5% among lichen planus patients in a period of 7.5 years, which is 50 times greater than its prevalence in the general population.^4^ On the other hand, infection with HCV is the most common etiologic factor for acute and chromic hepatitis worldwide. Hepatitis C becomes chronic in 75% of the cases and approximately 1-5% of the patients with hepatitis C develop primary hepatic cell carcinoma.^5^

The association between lichen planus and hepatitis C virus has been studied previously; however, the relationship between these two conditions has not been yet clearly established.^7^ Studies suggest that skin and mucosal lesions may be caused by direct action of the virus or by an induced immunological response, especially when erosive oral lesions are present. However, the data in medical literature vary much regarding prevalence of hepatitis C in patients with lichen planus.^8 - 10^

In the present study, means age differences between the groups were not statistically significant. No statistically significant differences were observed between the groups regarding distribution of males and females in the groups, consistent with other studies on lichen planus. In the present study, 60 patients with lichen planus (30 oral lichen planus patients and 30 cutaneous lichen planus patients) were evaluated considering the results of their HCV antibody test results. The results were compared with the same results from 30 healthy individuals, and there were no statistically significant differences between the groups. It was observed in the present study that buccal mucosa was the most common site for the lesions of the ulcerative type, and more patient have burning on nutrition, which is consistent with the results of a previous study, reporting the buccal mucosa as the most common site for oral lichen planus.^11^ In a study carried out to determine the prevalence of lichen planus in Hepatitis B and Hepatitis C patients, no relationship was found between oral lichen planus and Hepatitis B and C.^12^ However, of 23 cirrhotic patients, 2 patients had oral lichen planus lesions. Although this observation could be a sign of a relationship between the two diseases, no statistically significant association was found between oral lichen planus and Hepatitis C.

The results of the present study are consistent with the results of a study carried out on 505 patients with Hepatitis C in Spain,^13^ in which only 3.36% of the subjects had oral lichen planus, showing no statistically significant association. A study on 55 lichen planus patients in the Netherlands also failed to demonstrate such an association.^9^ The results of our study are also consistent with the results of another study on 66 oral and cutaneous lichen planus patients in Iran, in which only 1.5% of the patients had positive HCV antibody results, demonstrating no relationship between lichen planus and HCV infection.^6^ However, a study on 263 oral lichen planus patients in Italy revealed 28.8% positive HCV antibody test results, indicating a relationship between these two entities.^14^

Six different types and some subtypes of genotypes have been described (1a, 1b, 2a, 2b, 3, 4, 5a, 6a) for HCV. Genotype 1 is more frequent than non-1 genotypes (60% versus 40%). It could be suggested that the association between lichen planus and hepatitis C may result from infection with a particular form of HCV, a genotype found only in certain geographic areas. This may be the reason why many investigators have been unsuccessful in identifying a specific HCV genotype that could be responsible for the development of lichen planus.^14 , 15^

It is recommended to follow oral lichen planus lesions in Hepatitis C patients by a well-designed study. The results of such a study along with current literature can pave the way for conclusive results regarding the relationship between these two entities and determine indications for Hepatitis C serologic tests in oral lichen planus patients.

Conflicting results from different studies in different countries, including south European countries, and variations in immunologic factors such as HLA might explain the presence of relationship between lichen planus and HCV infection in some of the regions in the world.
